# Actin Dosage Lethality Screening in Yeast Mediated by Selective Ploidy Ablation Reveals Links to Urmylation/Wobble Codon Recognition and Chromosome Stability

**DOI:** 10.1534/g3.113.005579

**Published:** 2013-03-01

**Authors:** Brian Haarer, Lei Mi-Mi, Jessica Cho, Matthew Cortese, Susan Viggiano, Daniel Burke, David Amberg

**Affiliations:** * Department of Biochemistry and Molecular Biology, State University of New York (SUNY) Upstate Medical University, Syracuse, New York 13210; † Department of Biology, Syracuse University, Syracuse, New York 13244; ‡ Department of Biochemistry and Molecular Genetics, University of Virginia Medical Center, Charlottesville, Virginia 22908

**Keywords:** actin cytoskeleton, urmylation and elongator complexes, chromosome segregation, chromatid cohesion, synthetic dosage lethality

## Abstract

The actin cytoskeleton exists in a dynamic equilibrium with monomeric and filamentous states of its subunit protein actin. The spatial and temporal regulation of actin dynamics is critical to the many functions of actin. Actin levels are remarkably constant, suggesting that cells have evolved to function within a narrow range of actin concentrations. Here we report the results of screens in which we have increased actin levels in strains deleted for the ~4800 nonessential yeast genes using a technical advance called selective ploidy ablation. We detected 83 synthetic dosage interactions with actin, 78 resulted in reduced growth, whereas in 5 cases overexpression of actin suppressed the growth defects caused by the deleted genes. The genes were highly enriched in several classes, including transfer RNA wobble uridine modification, chromosome stability and segregation, cell growth, and cell division. We show that actin overexpression sequesters a limited pool of eEF1A, a bifunctional protein involved in aminoacyl-transfer RNA recruitment to the ribosome and actin filament cross-linking. Surprisingly, the largest class of genes is involved in chromosome stability and segregation. We show that actin mutants have chromosome segregation defects, suggesting a possible role in chromosome structure and function. Monomeric actin is a core component of the INO80 and SWR chromatin remodeling complexes and the NuA4 histone modification complex, and our results suggest these complexes may be sensitive to actin stoichiometry. We propose that the resulting effects on chromatin structure can lead to synergistic effects on chromosome stability in strains lacking genes important for chromosome maintenance.

Various yeast genome-wide screens have been developed that test pair-wise genetic interactions between hypomorphic or hypermorphic alleles. These screens have been particularly useful in uncovering genetic vulnerabilities and functional relationships between genes. Most widely recognized are whole genome synthetic lethal screens whereby double-mutant haploid strains are systematically constructed and analyzed for phenotype (Costanzo *et al.* 2010). Alternatively, we have developed whole-genome screening methods to identify deleterious combinatorial haploinsufficiencies (complex haploinsufficiency, or CHI) (Haarer *et al.* 2007, 2011). Synthetic lethal and CHI screens are examples of examining combinations of null and hypomorphic alleles. Others have exploited overexpression (hypermorphs) in combination with null alleles to identify synthetic dosage lethality (SDL) interactions (Measday *et al.* 2005) or synthetic dosage suppression interactions (Magtanong *et al.* 2011). The last two methods use modification of conventional synthetic genetic array screening to introduce overexpression plasmids into arrayed strains carrying deletion alleles of nonessential genes by genetic crosses. Few screens, however, take a whole-genome approach to examine the more subtle effects of approximately twofold overexpression of a particular gene, such as might occur with gene duplications, chromosomal translocations, or whole-chromosome aneuploidies (*e.g.*, Down syndrome). Although synthetic genetic array-modified SDL screening could be used with noninducible, low-copy-number plasmids, it requires that strains be put through meiosis (sporulation), followed by selection for desired, and counter-selection against nondesired, haploid progeny. Here we used a new method called selective ploidy ablation (SPA) (Reid *et al.* 2011) to screen for synthetic dosage interactions (SDIs) (Tong *et al.* 2004) with actin. SPA uses mating followed by chromosome destabilization to introduce plasmids into the haploid deletion collection strains.

The query of our modified SDI procedure is the essential actin gene of yeast. Actins are highly conserved in sequence and structure and are well known to polymerize to form thin 7-nm filaments that are organized by associated actin binding proteins into a diverse number of highly dynamic structures that are collectively called the actin cytoskeleton. The actin cytoskeleton is responsible for generating force and movement in cells. The actin cytoskeleton functions in endocytosis, exocytosis, polarized cell growth, cytokinesis, cell motility, translation (Kim and Coulombe 2010), as well as less well-defined functions in nuclear processes (Hofmann *et al.* 2004; Percipalle and Visa 2006) and as a component of chromatin remodeling complexes (Shen *et al.* 2000; Galarneau *et al.* 2000; Krogan *et al.* 2003). This functional complexity, sensitivity to gene dosage (Wertman *et al.* 1992), and dynamic behavior make actin a particularly rich target for genetic interaction analysis. We have previously reported two genome-wide screens for genetic interactions of actin. The first looked for CHI interactions between a null allele of actin and ~4800 null alleles for the nonessential genes, identifying ~240 deleterious CHI interactions (Haarer *et al.* 2007). This screen uncovered previously unappreciated functional relationships between actin and the ribosome, the Ccr4-Not transcriptional regulation complex, the ESCRT endocytic complexes, and the vacuolar ATPase, to name a few. A second screen used a modification of the procedure to examine the essential genes for CHI interactions with actin. This screen identified an additional 60 CHI interactions, and in this collection of genes there was tremendous functional enrichment, in particular for the proteasome and the TFIID complex (Haarer *et al.* 2011).

In this study we use SPA (Reid *et al.* 2011) to introduce a centromere plasmid carrying the actin gene into the ~4800 nonessential gene deletion collection. This procedure uses a donor strain that has galactose inducible promoters driving transcription across all 16 centromeres and a counter-selectable marker (*URA3*) linked to the 16 centromeres. This donor strain was transformed with the *ACT1* centromere plasmid, mated to deletion strains, chromosome loss was induced on galactose, and haploidized strains were selected on 5-fluoroorotic acid medium. The strains undergo a meiosis-independent transition from a 2N to 1N complement of chromosomes. The net result of this induced chromosome loss is to isolate haploid deletion strains containing the *ACT1* centromere plasmid. We identified 78 deleterious SDIs and five synthetic dosage suppression interactions. There was very high functional enrichment for genes that are novel and were not previously known to be directly related to actin function. Most prominently this included 14 genes involved in transfer RNA (tRNA) modifications (the elongator and urmylation pathways) that facilitate wobble position alternate base pairing and 19 genes involved in chromatid cohesion and/or chromosome segregation.

## Methods and Materials

### Plasmid and strain constructions

Yeast strains were grown on conventional media and strains were constructed by standard genetic methods (Amberg *et al.* 2005). Genome wide screens used robotics and methodologies described previously (Haarer *et al.* 2007). Strains ysed in this study are listed in Supporting Information, Table S1. The yeast gene deletion collection was obtained from EUROSCARF.

The nourseothricin resistance cassette (NAT^r^) was removed from plasmid p4339 (provided by C. Boone) as an ~1185-bp *Bam*HI-*Eco*RV fragment and cloned into *Bam*HI-*Sma*I-cut YCp50 and pKFW29, replacing the majority of the *URA3* gene in each; the resulting plasmids were named pLMM1 (*CEN*, *NAT*) and pLMM3 (*CEN*, *NAT*, *ACT1*), respectively. Yeast strain W8164-2B (Reid *et al.* 2011) was transformed with pLMM1 and pLMM3 to generate yeast strains LMMY1 and LMMY3, respectively.

### Robotic screening by selective ploidy ablation

Robotic screening was carried out by pinning the Euroscarf nonessential haploid deletion collection to YPD+G418 (200 µg/ml) and the query strains LMMY1 and LMMY3 to YPD+NAT (100 µg/ml). After 2-d growth at 25°, deletion strains were mated to each query strain by successively pinning each to YPD plates and incubating overnight at 25°. Mated strains were pinned to YPD+G418+NAT and incubated at 25° for 2 d to select for diploids. Destabilization of query strain chromosomes was induced by growth on YPGAL+G418+NAT for 2 d at 25°, after which, strains were pinned to SC+FOA+G418+NAT (NAT concentration was increased to 150 µg/mL in SC media) at 30° and 37°. Relative growth of strains from LMMY1- *vs.* LMMY3-based screens was noted after 2 to 4 d.

### Confirmation of SDIs

Sensitivity to extra *ACT1* was confirmed by independently transforming candidate deletion strains with YCp50 and pKFW29 (YCp50-*ACT1*) (Wertman *et al.* 1992). Strains were assessed for the subsequent abilities of transformants to grow at various temperatures on SC-ura medium. Growth of selected transformants were quantified by growing in liquid SC-ura and following OD_595_ using a TECAN Infinite 200 (Tecan Systems Inc., San Jose, CA) shaking incubator/spectrophotometer.

### Assays for chromosome loss

Actin alleles were integrated into a disomic chromosome VII strain 5912-SD4 after *Eco*RI digests of plasmids carrying *HIS3*-marked alleles of actin (Table S4) and transformants were selected on SC-his medium. These strains were grown in SC-lys-tyr medium to select for the disomes and 10^−2^, 10^−3^, and 10^−4^ dilutions were plated on YPD + cycloheximide medium to select for loss of heterozygosity for the *cyh2* allele. In addition, to determine cell viability, a 10^−4^ and 10^−5^ dilution was plated on YPD. White (*ade2 ade3*) and His^−^ (*ade3*) colonies were quantified on the cycloheximide plates and divided by the numbers of viable cells in each culture to determine rates of chromosome loss. Each strain was analyzed three independent times, average chromosome loss frequencies and standard deviations were calculated (Table S4).

## Results

### Primary robotic screens for synthetic dosage lethal interactions with actin

The *ACT1*/*CEN* plasmid pLMM3 and a negative control plasmid pLMM1 were transformed into the selective ploidy ablation host strain W8164-2B (Reid *et al.* 2011) and robotically mated to the Euroscarf haploid collection of null allele strains for the nonessential gene set. After diploid selection, the strains were robotically transferred to galactose-containing medium to induce transcription across the centromeres of the 16 chromosomes. Galactose-induced transcription across centromeres results in mitotic instability and can be used to induce 2N-1 aneuploidy (Hill and Bloom 1987; Reid *et al.* 2008). Cells were transferred to medium containing 5-fluoroorotic acid after growth on galactose to counter-select the *URA3* markers linked to the 16 centromeres derived from strain W8164-2B. Selection for the null allele on G418 media is maintained throughout to preclude loss of the null allele through destabilization of mitotically recombined chromosomes. The strains carrying the actin plasmid were scored manually/visually for colony size relative to control strains bearing pLMM1. In contrast to the methodology of Reid *et al.* (2011), we screened by using a 384-strain, rather than 1536-strain, format and used visual overlays to compare colony sizes. In our screen, we found that colonies were sufficiently irregular to prevent accurate digitization of colony size; this, coupled with more extensive edge-effects from a 384-strain format, necessitated manual analysis of data. We compared results from three independent screens and chose for further analysis those strains that displayed significant growth deficits relative to control in at least two of the screens.

### Confirming synthetic dosage sick or lethal interactions with actin

**Figure 1:**
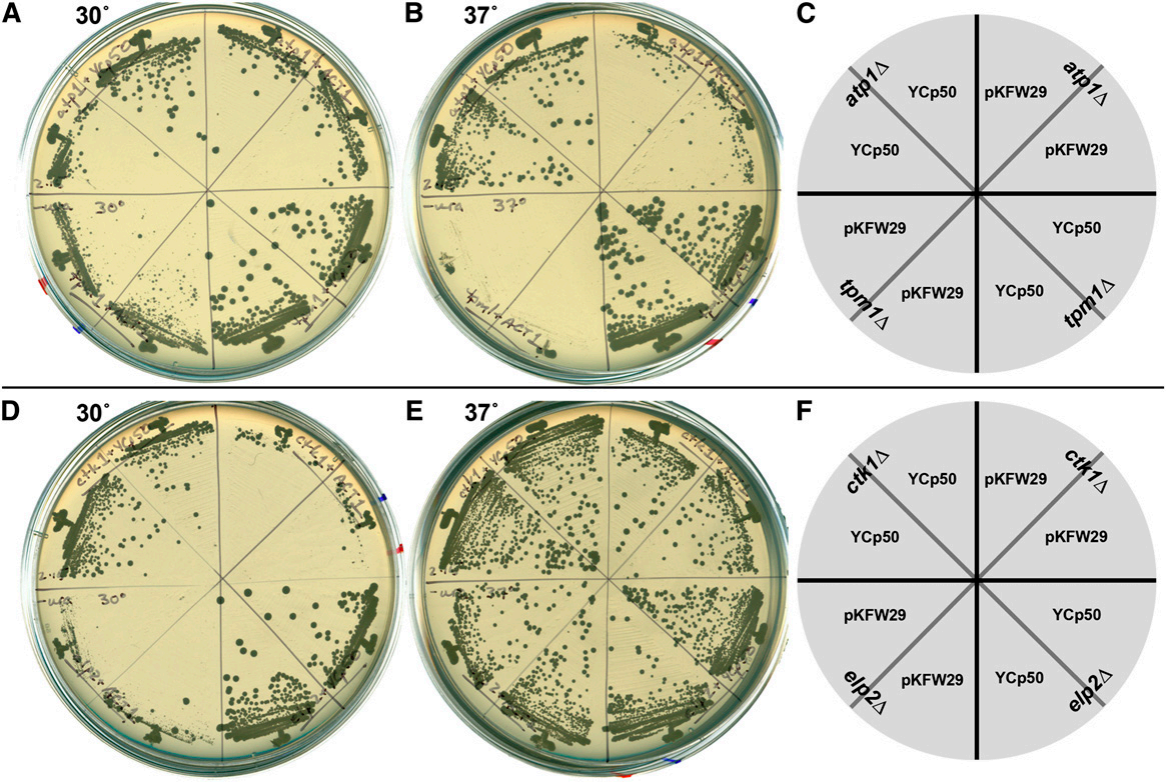
SDIs with actin. *atp1∆* and *tpm1∆* (A, B, and C), *ctk1∆* and *elp2∆* (D, E, and F) strains were transformed with control plasmid YCp50 or actin plasmid pKFW29 according to the keys (C and F), the resulting transformants were struck for single colonies, and grown at 30° (A and D) or 37° (B and E).

The YCp-*ACT1* plasmid and YCp50 were transformed in parallel into haploid deletion strains and the transformations were scored for both transformation efficiency and colony size upon restreaking. Figure 1 shows examples of plasmid-containing strains identified by robotic screening that showed a range of severity of growth phenotypes in the presence of the YCp-*ACT1* plasmid. The *atp1∆* strain had a modest growth defect at 37° (Figure 1B), *tpm1∆* had severe growth defects at both temperatures (Figure 1, A and B), whereas *ctk1∆* and *elp2∆* had growth defects only at 30° (Figure 1, D and E). Strains having the strongest interactions in the transformation assay were reproducibly identified in the robotic screens. Confirmation tests led to the identification of 25 synthetic dosage lethal or synthetic dosage sick interactions. Note that we collectively refer to these negative genetic interactions as SDL interactions.

We examined published data and data available from The *Saccharomyces* Genome Database (http://www.yeastgenome.org/) concerning the functions of the genes showing SDI with actin and derived a list of functionally related genes we might expect to show SDIs with actin (the implicated genes). We tested an additional 136 genes by using the transformation-based procedure described previously and found that 58 show an SDI (either negative or positive) with actin. The implicated list was more enriched for actin SDIs than the entire nonessential gene deletion set, suggesting functional enrichment in genes that show SDI with actin.

### Synthetic dosage suppression interactions

**Figure 2:**
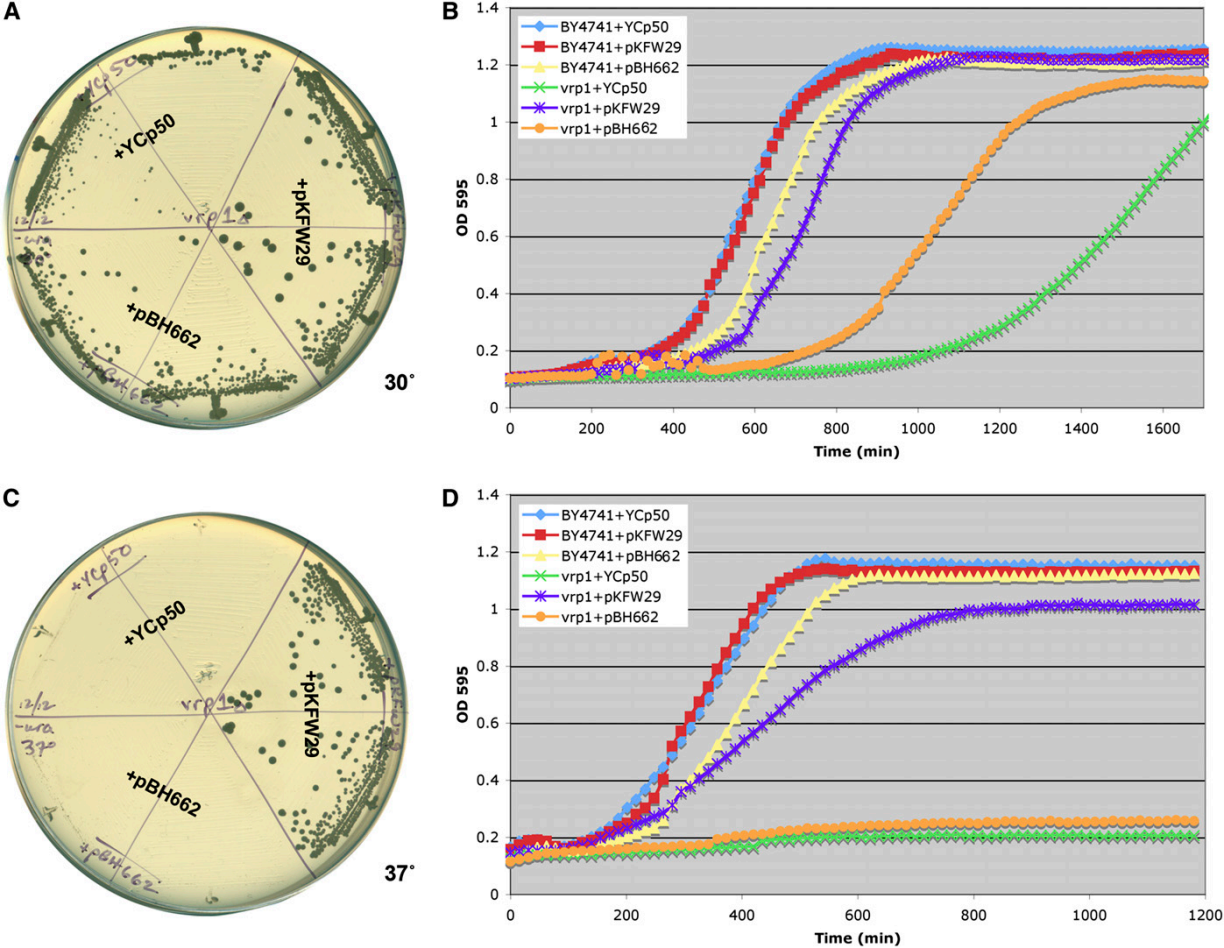
Extra monomeric actin suppresses the loss of verprolin. Wild-type yeast strain BY4741 or a *vrp1∆* strain were transformed with the empty control plasmid YCp50, the YCp-*ACT1* plasmid pKFW29, or the YCp-*act1-159* plasmid pBH662. *vrp1∆* transformants were struck on SC-Ura plates and incubated at 30° (A) or 37° (C). BY4741 (wild-type) and *vrp1∆* transformants were inoculated into SC-Ura liquid medium and growth rates were monitored in a TECAN microplate reader at 30° (B) or 37° (D).

Although the largest class of interactions identified was negative interactions, a small number of synthetic dosage suppression interactions (high copy suppression) were identified. The strongest suppression was seen with a deletion of the gene for verprolin a yeast ortholog of human WASP interacting protein or WIP. A *vrp1∆* strain is unable to grow at 37°, and this growth defect can be completely suppressed by an extra copy of the actin gene (Figure 2, C and D). Suppression of the *vrp1-1* allele by extra actin has been previously reported (Vaduva *et al.* 1997). The *vrp1∆* strain also grows less well at 30° than a wild-type strain, and this defect is also suppressed by extra actin (Figure 2, A and B). Interestingly, an actin filament stabilizing form of actin encoded by the *act1-159* allele (Belmont and Drubin 1998) has little-to-no ability to suppress *vrp1∆* (Figure 2; *act1-159* is carried on plasmid pBH662). Verprolin is reported to be an actin monomer binding protein that also interacts with the Arp2/3 activator Las17 (WASP) and the cytokinesis regulator Hof1p (Munn and Thanabalu 2009). Verprolin acts as an actin monomer chaperone for actin filament assembly (Munn and Thanabalu 2009), and we propose that loss of verprolin can be suppressed by increasing the amount of monomeric actin but not filamentous actin.

We also observed suppression of the growth defects caused by deletion of *GIM3* and *PAC10*, two genes for components of the prefoldin complex. Deletions of several genes for proteins of the prefoldin complex were complex haploinsufficient with an actin deletion, including *gim3∆* and *pac10∆* (Haarer *et al.* 2007). The prefoldin complex acts as a chaperone delivering unfolded actin and tubulin to the CCT chaperonin complex for folding (Vainberg *et al.* 1998). Interestingly, *act1∆* was also complex haploinsufficient with deletion of two genes for CCT complex members, *cct4∆* and *cct8∆* (Haarer *et al.* 2011). Collectively, these results are consistent with mass-action effects on actin folding: (1) When actin levels are low simultaneous reductions in prefoldin or CCT complex levels leads to pools of folded actin that are inadequate to sustain cell viability, and (2) Under conditions of reduced folding capacity, such as in prefoldin deletion mutants, folded actin becomes limiting, affecting growth rate, but increasing actin expression can push the equilibrium such that levels of folded actin become adequate to restore normal growth rates.

Finally, reduced growth rates in *sac6∆* and *taf14/anc1∆* could be partially suppressed by actin overexpression. Sac6 cross-links and stabilizes actin filaments within the actin cortical patch (Goodman *et al.* 2002) and we propose that in its absence, an increased actin concentration can partially compensate for the loss of this activity. Anc1/Taf14 is reported to be in a number of protein complexes that are involved in transcription and chromatin remodeling, some of these complexes also contain actin (*e.g.*, the INO80 complex (Shen *et al.* 2000)). *anc1∆/taf14∆* is CHI with *act1∆* suggesting that Anc1p/Taf14p-containing complexes are sensitive to actin stoichiometry.

### The actin SDI gene network

We have found 78 genes that display SDL interactions and five genes whose deletions are partially suppressed by an extra copy of the actin gene (Table S2). It is unlikely that we have identified all of actin’s SDIs for the nonessential gene set. There is some overlap between the nonessential genes that showed a CHI interaction with actin and the genes that show SDI with actin; these include *CHS1*, *CNM67*, *SPC72*, *GIM3*, *PAC10*, *BEM2*, *VMA4*, *VMA7*, *VMA8*, *VMA9*, *VMA10*, *VMA13*, *SAC2/VPS52*, *RSM19*, and *SHP1*. Loss of these genes causes sensitivity to both elevated and decreased actin stoichiometry. The actin SDI network is highly enriched for specific functional categories. Table S2 shows a list of all genes identified in our SDI screen. Based on broad functional relatedness, the genes are displayed in groups. The first group, shown in green, comprises genes that in some way are related to a specific set of tRNA uridine modifications that allow for wobble position base-pairing during translation. The second category of genes, displayed in red, is functionally related to chromatid cohesion, chromosome segregation, and microtubule function. Members of the third group (shown in blue) are involved in actin function, the cell cycle, and cell growth. We also identified eight genes for vacuolar ATPase subunits (*VMA2*, *VMA4*, *VMA7*, *VMA8*, *VMA9*, *VMA10*, *VMA13*, and *VMA16*), two of the three genes that encode for subunits of the kinase that phosphorylates the C-terminal domain of RNA Polymerase II (*CTK1* and *CTK2*), and one gene involved in endosome trafficking (*SAC2*).

### The elongator complex, urmylation, and tRNA wobble codon base pairing

The Funspec web site (http://funspec.med.utoronto.ca/) was used to determine whether there was statistically significant functional enrichment in the SDI network (Table S3). The most significant enrichment (*P* = 1 × 10^−14^) was for the Gene Ontology Biological Process “tRNA wobble uridine modification.” We identified most of the genes for two pathways that catalyze two modifications on the wobble anti-codon uridine tRNA U34. The “elongator holoenzyme complex” (*P* value for enrichment = 6.9 × 10^−10^) is encoded by the genes *ELP2*, *ELP3*, *ELP4*, *ELP6*, and *IKI3* and catalyzes the cm5 (5-carboxymethyl) moiety of the modifications ncm^5^U (5-carbamoyl-methyuridine), cm^5^U (5-carboxymethyluridine), and mcm^5^U (5-methoxycarbonylmethyluridine) (Jablonowski and Schaffrath 2007). The product of *TRM9* gene that was also identified in our screen catalyzes the formation of mcm^5^U and cm^5^U (Huang *et al.* 2008). The protein urmylation pathway (*P* = 1.47 × 10^−14^) is encoded by the genes *NCS6*, *ELP2*, *UBA4*, *URM1*, *ELP6*, *NCS2*, and *URE2* and forms a sulfur relay system that is responsible for modifying tRNA U34 at position 2 by thiolation (Noma *et al.* 2009). Some of the elongator complex proteins are also required for the thiolation modification. Urm1, which participates in the sulfur relay reaction, is related in sequence to ubiquitin (Hochstrasser 2009) and can be conjugated to the lysine residues of proteins; only a few substrates have been identified (Van der Veen *et al.* 2011), but one of them, the hydroperoxidase Ahp1, was also identified as showing an SDI with actin.

**Figure 3:**
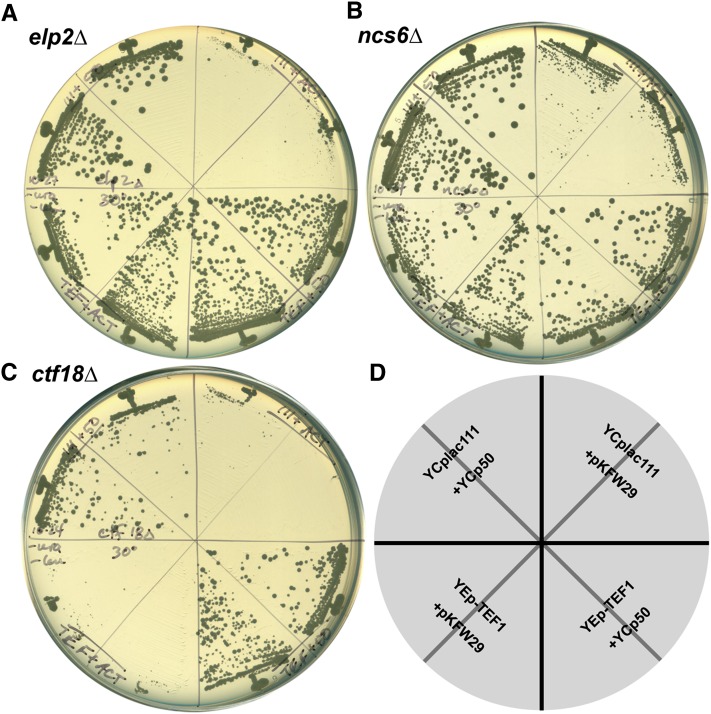
Tef1p overexpression suppresses the actin SD interactions with *elp2∆* and *ncs6∆* but not *ctf18∆*. An *elp2∆* strain (A), an *ncs6∆* strain (B), and a *ctf18∆* strain (C) were transformed with the indicated combinations of control plasmids YCplac111 and YCp50, or actin expression plasmid pKFW29 and Tef1p overexpression plasmid YEp-*TEF1*. Transformants were streaked on plates according to the key (D) and incubated at 30°C.

One interpretation of these data is that the SDI with these mutants reflect an impact of actin overexpression on translation efficiency. Many observations have suggested a role for the actin cytoskeleton in translation (Kim and Coulombe 2010) and translation elongation factor 1A (eEF1A) is known to be an actin binding and cross-linking protein (Gross and Kinzy 2005). We asked whether overexpression of eEF1A (encoded by the paralogs *TEF1* and *TEF2*) affects the SDI of actin with elongator and urmylation gene deletions (Figure 3). Remarkably, overexpression of Tef1p completely suppressed the actin SDI with *elp2∆* (Figure 3A) and *ncs6∆* (Figure 3B) but had no effect on the SDI with *ctf18∆* (Figure 3C). The product of the *CTF18* gene is involved in sister chromatid cohesion and has no known role in translation. To confirm specificity of the eEF1A suppression to the Elp and Urm pathways, we tested several additional mutants. We found that the *elp6∆*, *urm1∆*, and *ncs2∆* SD interactions with actin were suppressed by eEF1A overexpression whereas the *bim1∆*, *kar3∆*, *swi4∆*, *ctk1∆*, *msy1∆*, and *luv1∆/vps54∆* SD interactions were not. The only exception with respect to specificity for genes involved in wobble-U modification was suppression of the *chs7∆* SD interaction by eEF1A overexpression. However, the function of *CHS7* is not known and so we currently do not know the mechanism by which eEF1A overexpression suppresses the actin *chs7∆* SD interaction. These results suggest that the actin cytoskeleton and translation compete for a limited pool of eEF1A (see *Discussion*).

### The SDI with *ssk1∆* was due to a background mutation in *CHL1*

The *ssk1∆* strain from the deletion collection was quite sensitive to actin overexpression, especially at 37°. We could not confirm the interaction using the independently derived *ssk1∆* strain BBY181. Backcrossing and polymerase chain reaction of strains from different genetic backgrounds indicated that the Euroscarf strain carries a *bona fide ssk1∆* allele but that there is a second mutation in the strain that is required for the sensitivity to actin overexpression. In crosses of the Euroscarf strain, we observed little second-division segregation of the second mutation, indicating that the other gene was centromere linked. To identify this second gene, we isolated several clones, after transformation with a genomic (YCp) library, that were able to complement the temperature sensitivity associated with the combination of actin overexpression and the second mutation. Sequencing revealed that several of these suppressing clones encompass overlapping fragments that contain genes linked to the centromere of chromosome XVI. Subcloning and retesting indicated that the second mutation in the Euroscarf *ssk1∆* strain is in *CHL1*, a probable DNA helicase involved in sister-chromatid cohesion and genome integrity (*Saccharomyces* Genome Database; www.yeastgenome.org). *CHL1* is related to the human ChlR1 helicase of similar function and to the BRCA1-binding helicase FANCJ (Wu *et al.* 2009). Polymerase chain reaction-based recovery and sequencing of the mutant *chl1* locus revealed a single mutation in the 2586-bp coding region that results in changing the highly conserved (among Chl1p orthologs) glycine 548 to glutamate. This position corresponds to Gly569 at/near the C-terminal boundary of the Arch domain of the FANCJ/XPD family of helicases; this domain is proposed to wrap around single-stranded DNA (Fan *et al.* 2008; Lehmann 2008; Liu *et al.* 2008; Wu *et al.* 2012). The *ssk1∆* allele present in the deletion strain does not appear to contribute to the pKFW29 sensitivity of strains carrying the *chl1^G548E^* mutation. We found that the Euroscarf *chl1∆* strain is just as sensitive to actin overexpression as strains carrying the *chl1^G548E^* allele (Figure S1). Our fortuitous identification of *chl1* led us to examine genes of related function, resulting in an expanded list of genes that display SDI with actin (Table S2, genes in red).

### Chromatid cohesion, chromosome segregation, and microtubule-related functions

The second large cluster of functionally related genes identified in the screen had functions related to chromosome dynamics during mitosis. We identified SDI with genes involved in three broadly defined processes of genome stability, sister chromatid cohesion (*DCC1*, *RAD61*, *CTF8*, *CTF18*, *TOF1*, *CHL1*, *CTF4*), kinetochore function (*BIM1*, *IML3*, *CHL4*, *MCM21*, *MCM22*, *CTF3*, *CHL1*, *CTF19*, *MCM16*), mitotic motors (*KAR3*, *CIK1*) and spindle pole body function (*SPC72*, *CNM67*). Strikingly, these genes that regulate genome stability are thought to function primarily in mitosis (Winey and Bloom 2012), and most of the genes encode nuclear proteins. The two spindle pole proteins are an exception as Cmn67 resides in the outer (cytoplasmic) plaque of the spindle pole body and Spc72 is a component of the cytoplasmic gamma tubulin complex (Helfant 2002).

The SDI with a large number of nuclear proteins required for chromosome segregation is puzzling, given the conventional view that actin is a cytoplasmic protein. However, actin has been shown to be a component of the INO80 chromatin remodeling complex (Shen *et al.* 2000), the NuA4 histone acetyltransferase complex (Galarneau *et al.* 2000), and the SWR chromatin remodeling complex (Krogan *et al.* 2003) and therefore actin is clearly in a position to influence the structure and function of chromosomes. Furthermore, all three of these complexes have been found to associate with the kinetochores, and mutations in Arp4, a shared component of these complexes, cause defects in kinetochore assembly (Ogiwara *et al.* 2007). In addition, human Arp8, a component of INO80, localizes to mitotic chromosomes and its depletion results in chromosome misalignment during mitosis (Aoyama *et al.* 2008).

**Figure 4:**
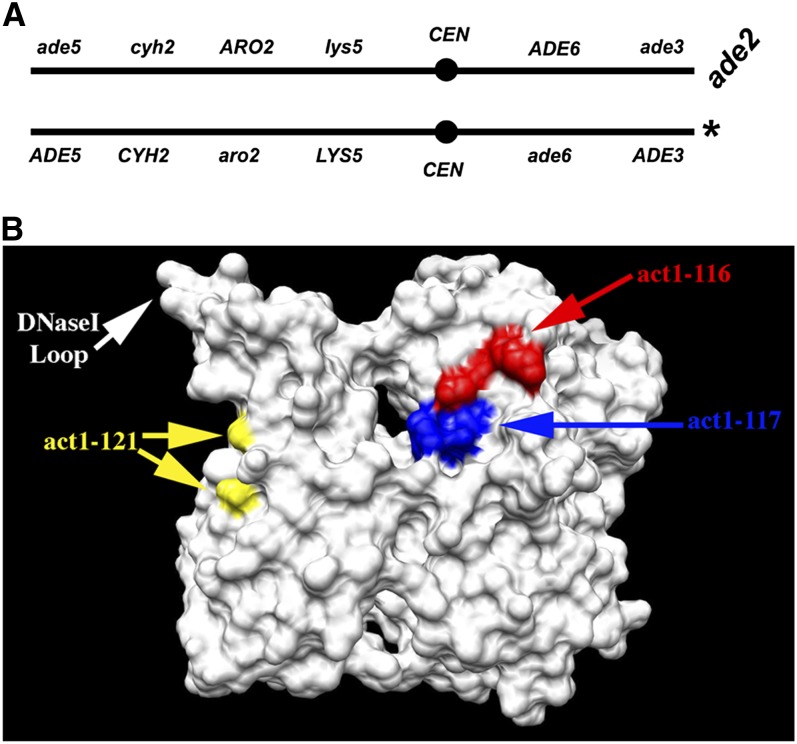
Mutations in conventional actin lead to elevated levels of chromosome loss. (A) Diagram of the two copies of chromosome VII in strain 5912-SD4 used to measure the frequencies of chromosome loss in actin mutants. Recessive, loss-of-function alleles of genes are indicated in lower case whereas wild-type alleles of genes are indicated in upper case. In addition, the strain has a loss of function allele of the *ade2* gene. Loss of the wild-type *CYH2* allele by loss-of-heterozygosity can be selected for by cycloheximide resistance, which can occur by gene conversion, mitotic recombination, or chromosome loss. Loss of the chromosome indicated by an asterisk leads to cycloheximide resistant, white colonies (*ade2 ade3*) that require histidine (his^-^ due to the *ade3* allele). (B) The location of mutated residues in actin that cause chromosome loss. The solvent exposed surface of yeast actin was rendered with the program Chimera (http://www.cgl.ucsf.edu/chimera) using coordinates from the PDB file 1YAG.pdb (Vorobiev *et al.* 2003). The side-chains of residues mutated by allele *act1-116* are rendered in red, the side chains of residues mutated by allele *act1-117* are rendered in blue, and the side chains of residues mutated by *act1-121* are rendered in yellow. Note that the surface shown is largely buried within an actin filament.

To determine whether actin has a role in chromosome segregation we analyzed a set of actin alanine scan mutants (Wertman *et al.* 1992) for rates of chromosome loss in strains disomic for chromosome VII. Figure 4A is a graphical representation of the disomic chromosome VII used to score chromosome loss in the actin mutants. The disomic chromosome VII is heterozygous for several alleles that can be used to select for chromosome instability events and distinguish between chromosome loss, mitotic recombination and gene conversion. The actin alleles (Table S4) were integrated into the disomic strain and cells were grown in medium selecting for the disome and plated on medium containing cycloheximide. Resistance to cycloheximide requires loss of the wild-type *CYH2* allele, which can occur by gene conversion, mitotic recombination, or loss of the chromosome indicated with an asterisk in Figure 4A. Colonies that arose due to chromosome loss were white (*ade3^−^ ade2^−^*) and his^−^ (*ade3^−^*). The results of this analysis are in Table S4. Three of the alleles were found to cause an ~10-fold increase in chromosome loss: *act1-116* (D187A, K191A), *act1-117* (R183A, D184A), and *act1-121* (E83A K84A). Figure 4B shows the location of the mutated residues encoded by these alleles on a surface rendering of the yeast actin molecule (Vorobiev *et al.* 2003). The most notable aspect of these data are that the mutated residues would be buried in an actin filament and therefore inaccessible to actin filament binding proteins. Therefore, if these residues identify sites of protein-protein interaction, they would most likely indicate binding to an actin monomer, as this surface would only be accessible within the monomer. Interestingly, there is no evidence that the actin found in chromatin remodeling complexes is in the filamentous form, quite to the contrary, the stoichiometries are most consistent with the actin being in the monomer state (Shen *et al.* 2000; Galarneau *et al.* 2000; Krogan *et al.* 2003; Fenn *et al.* 2011). It is possible that these mutations identify a binding interface with Arp4p, which is known to interact directly with actin within chromatin remodeling and modification complexes (Fenn *et al.* 2011).

## Discussion

Network analysis of whole-genome genetic interaction data from yeast indicates a small-world network with short path lengths between genes and processes (Boone *et al.* 2007). This suggests a level of integration between cellular systems that we are just beginning to appreciate but with little understanding of the molecular mechanisms of integration. Our previous studies performing CHI screens with actin identified large numbers of genes (~300) whose functions were enriched for a number of core cellular processes (Haarer *et al.* 2007, 2011), which suggests that the actin cytoskeleton may be particularly important for facilitating cross-talk and integration between cellular functions. The actin cytoskeleton is well suited to such a role as actin is multifunctional, is found in a diverse number of protein complexes interacting with a large number of proteins, and is found in both the cytoplasmic and nuclear compartments. Furthermore, actin can exist in a number of different states of organization that change rapidly in response to environmental change and stress. An excellent example of a possible integrating role of actin is suggested by our recent observations showing that actin disruption can impact proteasome function and *vice-versa* and that these two protein complexes interact directly. This functional coupling was first suggested by CHI interactions between a null allele of actin and null alleles of many proteasome subunit genes (Haarer *et al.* 2011).

Prominent in our CHI screens has been the identification of genes encoding core components of the translation apparatus, in particular ribosomal subunit genes (Haarer *et al.* 2007). Ribosomes are associated with actin filaments *in vivo* (Wolosewick and Porter 1976; Lenk *et al.* 1977; Wolosewick and Porter 1977), and disruption of the actin cytoskeleton in yeast leads to a reduction in protein synthesis (Kandl *et al.* 2002; Gross and Kinzy 2007). One component of the translation apparatus, eEF1A, has been shown to bind and cross-link actin filaments and to play a role in proper actin organization in yeast (Gross and Kinzy 2005). eEF1A’s role in translation is to recruit aminoacyl-tRNAs to the ribosome during translation and eEF1A binding to aminoacyl-tRNAs is inhibited by F-actin binding (Liu *et al.* 1996). These results suggest that eEF1A mediates cross-regulation between the actin cytoskeleton and protein synthesis. One of the largest groups of genes identified in this SDI study includes genes for the urmylation pathway and elongator complex proteins, which have an indirect role in translation by modifying the wobble uridine in the anti-codon loop of some tRNAs (Jablonowski and Schaffrath 2007; Noma *et al.* 2009). The negative SDIs caused by actin overexpression in strains deleted for urmylation and elongator pathway genes can be suppressed by overexpressing eEF1A. We propose that eEF1A is limiting in cells and this pool is partitioned between binding to the actin cytoskeleton and being free to deliver aminoacyl-tRNAs to the ribosome. Overexpression of actin creates more binding sites for eEF1A, leaving less eEF1A free to participate in translation elongation. When combined with defects in tRNA uridine wobble anticodon modification, cumulative reductions in translation elongation rates result in inadequate rates of protein synthesis and reductions in cell growth and viability. Interestingly, most proteins involved in translation are very abundant in cells but two reports suggest that eEF1A may be present in only a few hundred copies (Ghaemmaghami *et al.* 2003; Huh *et al.* 2003). However, an earlier report claims that eEF1A is an abundantly expressed protein (Thiele *et al.* 1985) and so the expression level of eEF1A remains unresolved at this time. Regardless, we theorize that eEF1A levels evolved to be limiting as a mechanism for cells to coordinate protein synthesis with the state of the actin cytoskeleton; under conditions that cause F-actin disassembly bulk protein synthesis would be increased while under conditions that cause enhanced F-actin assembly, protein synthesis would be reduced. It was recently reported that decreased levels of eEF1A can increase the rapid tRNA decay pathway and thus reduce the levels of tRNAs in the cell (Dewe *et al.* 2012). Therefore, increased sequestration of eEF1A on actin filaments could also be affecting rates of protein translation through reductions in tRNA levels.

Previous genetic screens with actin have uncovered a number of connections to processes that regulate chromatin. In one of the first noncomplementation screens performed in yeast, alleles of *ANC1* (Actin Non-Complementing) were found to not complement the *act1-1* allele of actin (Welch *et al.* 1993). *ANC1* was also identified in our screen for complex haploinsufficiencies (a variation of a noncomplementation screen using null alleles) with an *act1∆* allele (Haarer *et al.* 2007). Anc1p/Taf14p, as well as actin, has since been found to be a core component of the INO80 chromatin remodeling complex (Shen *et al.* 2000, 2003). Our CHI screen with *act1∆* also identified deletion alleles of genes for three other components of INO80 including *ARP8*, *IES1*, and *IES3* (Haarer *et al.* 2007) and the SDI screen described here identified *IES4*. In addition, the CHI screen identified genes for three components of the NuA4 histone acetylase complex including *EAF3*, *EAF5*, and *EAF6*. Actin is also known to be a core component of the NuA4 complex (Galarneau *et al.* 2000). Actin and the actin-related protein Arp4 comprise a shared core module of not only the INO80 and NuA4 complexes but also the SWR chromatin remodeling complex (Krogan *et al.* 2003). We propose that these complexes and the processes they regulate are sensitive to actin gene dosage and this is the basis for our identification of genes involved in chromosome segregation in the SDI and CHI screens.

The INO80, SWR, and NuA4 complexes all share a common influence on chromatin composition by regulating the localization of histone H2A.Z-containing nucleosomes (Htz1p in yeast). NuA4 has been shown to acetylate histone H4, and is required to recruit the SWR chromatin remodeling complex. The function of SWR is to exchange histone H2A for histone H2A.Z in discreet regions of the genome such as at double-strand breaks, promoters, heterochromatin-euchromatin boundaries, and centromeric DNA (Lu *et al.* 2009). The INO80 complex on the other hand opposes this activity by replacing H2A.Z with H2A; in INO80 mutants H2A.Z becomes mis-localized through-out the yeast genome (Watanabe and Peterson 2010). The H2A *vs.* H2A.Z composition of histones locally affects the structure, composition and therefore activity/function of that region of the genome. Most relevant to the screen presented here are observations that Htz1p recruitment to centromeres is dependent on both the NuA4 and SWR complexes and that deletion of *HTZ1*, and genes for components of both the SWR and NuA4 complexes, result in chromosome segregation defects comparable to those we report here for actin mutants (Krogan *et al.* 2004).

All reports for the composition of the INO80, NuA4, and SWR complexes indicate that actin is present as a monomer (Galarneau *et al.* 2000; Shen *et al.* 2000; Krogan *et al.* 2003; Fenn *et al.* 2011). Over the years, the presence of nuclear actin has been controversial in part because it has been difficult to detect in the nucleus even with fluorescently labeled, high affinity F-actin binding reagents such as phalloidin. Perhaps nuclear actin functions as a monomer and its levels are kept low to prevent polymerization from interfering with its interactions with components of nuclear complexes such as INO80, NuA4, and SWR. Arp4p and Arp8p have been shown to interfere with actin polymerization *in vitro* (Fenn *et al.* 2011), suggesting that actin-actin interactions within actin filaments are exclusive to actin-Arp interactions. We theorize that our actin CHI screen identified genes for components of the INO80 and NuA4 complexes because nuclear actin levels are too low to adequately populate these complexes. Furthermore, we suggest that the SDI screen with actin identified so many genes involved in chromosome segregation because nuclear actin has been raised to such a level that actin-actin interactions are interfering with the actin-Arp4 interaction required for the functions of INO80, NuA4, and SWR complexes. The result is misregulated deposition of Htz1p at centromeres and cumulative and toxic levels of chromosome missegregation.

Using SPA for performing dosage screens has limitations, as the overlap between screens shows that the procedure produced significant numbers of false positives and negatives. The advantages of the SPA procedure is that it allows any lab to introduce plasmids into haploid yeast collections without requiring the liquid robotics that are typically not available to most labs. While not exhaustive on its own, the SPA procedure is very effective for identifying relevant pathways for follow-up analysis.

In summary, the results presented here and in our previous CHI screens suggest that many cellular pathways that involve actin have evolved to operate within a narrow range of actin concentrations coupled to a finely tuned balance between the pools of monomeric *vs.* filamentous actin. A likely consequence of this situation is that many cellular processes can be coordinately regulated by stresses that affect the G- to F-actin ratio; some processes may be sensitive to excessive actin monomer or excessive actin filaments, while others may be sensitive to limitations in actin monomer or actin filaments.

## Supplementary Material

Supporting Information
