# Supplementary material for: Actin Dosage Lethality Screening in Yeast Mediated by Selective Ploidy Ablation Reveals Links to Urmylation/Wobble Codon Recognition and Chromosome Stability
Source: G3 (Bethesda). 2013 Mar 1;3(3):553–61. doi: 10.1534/g3.113.005579 (PMC3583461; doi:10.1534/g3.113.005579)
Supplement: Supporting Information [file supp_3_3_553__index.html]

Supporting Information 

# Actin Dosage Lethality Screening in Yeast Mediated by Selective Ploidy Ablation Reveals Links to Urmylation/Wobble Codon Recognition and Chromosome Stability

## Supporting Information for Haarer *et al.*, 2013

**Files in this Data Supplement:**

- Supporting Information - Figure S1 and Tables S1-S4 (PDF, 1.9 MB)
- Figure S1 - Actin over-expression is toxic to *chl1∆* cells (PDF, 1.8 MB)
- Table S1 - Yeast Strains (PDF, 69 KB)
- Table S2 - Synthetic dosage interactions with actin (.xls, 32 KB)
- Table S3 - Functional enrichment in the actin SDI network (.xls, 11 KB)
- Table S4 - Actin alanine scan mutants that cause chromosome segregation defects (.xls, 44 KB)
