# Supplementary material for: Actin Dosage Lethality Screening in Yeast Mediated by Selective Ploidy Ablation Reveals Links to Urmylation/Wobble Codon Recognition and Chromosome Stability
Source: G3 (Bethesda). 2013 Mar 1;3(3):553–61. doi: 10.1534/g3.113.005579 (PMC3583461; doi:10.1534/g3.113.005579)
Supplement: Supporting Information [file supp_3.3.553_005579SI.pdf]

G3 · Genes | Genomes | Genetics

Actin Dosage Lethality Screening in Yeast Mediated by Selective Ploidy Ablation Reveals Links to Urmylation/Wobble Codon Recognition and Chromosome Stability

B. Haarer[1], Lei Mi Mi[1], J. Cho[1,2], M. Cortese[1], S. Viggiano[1], D. Burke[3], and D. Amberg[1]

[1]Department of Biochemistry and Molecular Biology, State University of New York (SUNY) Upstate Medical University, Syracuse, NY 13210; [2]Department of Biology, Syracuse University, Syracuse, NY 13244; [3]Department of Biochemistry and Molecular Genetics, University of Virginia Medical Center, Charlottesville, VA 22908

[*] Corresponding Author:

David C. Amberg
Department of Biochemistry and Molecular Biology
SUNY Upstate Medical University
750 E. Adams St.
Syracuse, NY 13210
(315)464-8727
ambergd@upstate.edu

DOI: 10.1534/g3.113.005579

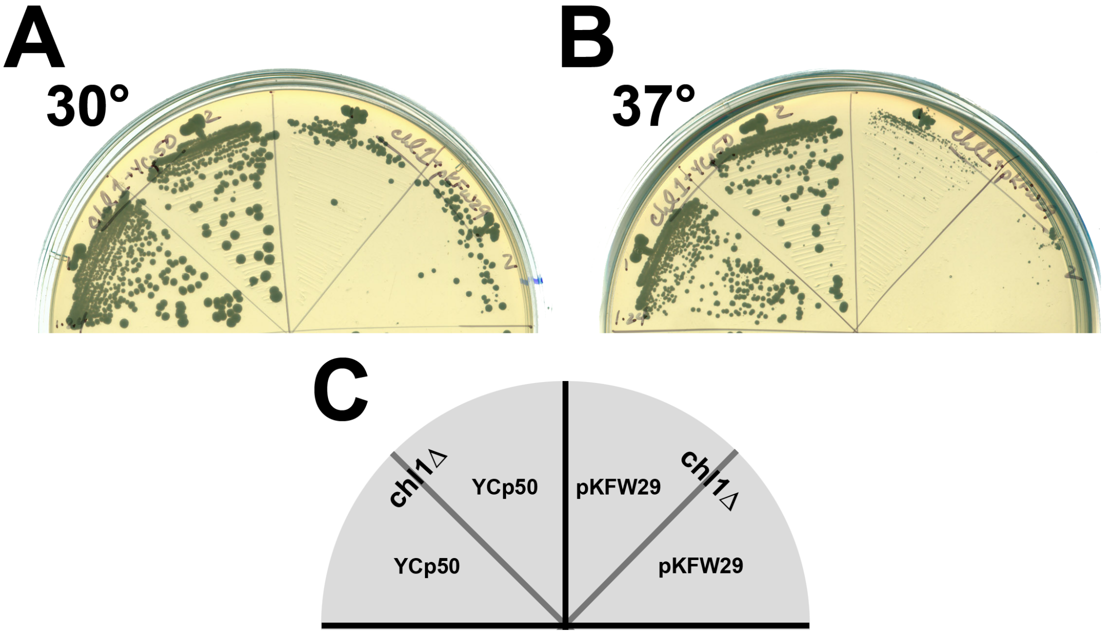

Figure S1 Actin over-expression is toxic to *chl1Δ* cells. A *chl1Δ* strain was transformed with the control plasmid YCp50 or the actin expression plasmid pKFW29, the transformants were streaked on plates according to the key (C) and incubated at 30°C (A) and 37°C (C).

Table S1 Yeast Strains. All strains used in this study were isogenic with BY4741 except W8164-2B (W303), BBY181, LMMY1 and LMMY3 (S288C) and 5903SD-3 (A364a).

Name	Source	Genotype
W8164-2B	R. Rothstein	*MATa CEN1GCS CEN2GCS CEN3GCS CEN4GCS CEN5GCS CEN6GCS CEN7GCS CEN8GCS CEN9GCS CEN10GCS CEN11GCS CEN12GCS CEN13GCS CEN14GCS CEN15GCS CEN16GCS ADE2 can1-100 his3-11,15 leu2-3,112 LYS2 met17 trp1-1 ura3-1 RAD5*
LMMY1	This study	W8164-2B + [pLMM1]
LMMY3	This study	W8164-2B + [pLMM3]
BY4741	SGD	*MAT***a** *his3Δ1 leu2Δ0 ura3Δ0 met15Δ*
5903SD-3	D. Burke	*MATα ade5/+ lys5-H1/+ cyh2/+ +/aro2-H1 leu1-1/+ trp5-H1/+ +/ade6 ade3/+ ade2-1 his3 Δ1 ura3-52 trp1-289 leu2-3,112 can1*
BBY181	This study	*MAT***a** *ura3-52 his3Δ200 leu2Δ1 trp1Δ63 ssk1Δ::hgh*

Tables S2-S4 are available for download at http://www.g3journal.org/lookup/suppl/doi:10.1534/g3.113.005579/-/DC1

Table S2: Synthetic dosage interactions with actin. The first column lists genes whose null alleles were found to be either sensitive or suppressed by actin over-expression. The second column indicates if the gene was hit in the primary robotic screens (1°) or if it was implicated by an actin SDI interaction with a functionally related gene, and if so, what the name of that gene is. The 3rd, fourth and fifth columns summarize functional information about the encoded gene products as ascertained from the *Saccharomyces* Genome Database (http://www.yeastgenome.org/). Three large functional groups are color coded: green for functions related to tRNA wobble anti-codon uridine modification, red for functions related to chromosome transmission and fidelity, and blue for functions related to cell growth and division. The 6th column indicates the temperatures at which an SDI interaction was observed. All interactions can be assumed to be negative unless suppression was observed which is indicated by SDS for synthetic dosage suppression.

Table S3: Functional enrichment in the actin SDI network. The genes listed in Table 2 were analyzed with the web-based tool Funspec (http://funspec.ccbr.utoronto.ca/) to identify functional categories based on GO (**G**ene **O**ntology) terms and MIPS terms (data compiled by the Munich Information Center for Protein Sequences (http://mips.helmholtz-muenchen.de/genre/proj/yeast/)) that are enriched in the SDI network of genes. The most significantly enriched aspects are reported in the second column with the names of the genes from the network that contribute to this enrichment listed in column four.

Table S4: Actin alanine scan mutants that cause chromosome segregation defects. The indicated actin alanine scan alleles were integrated into the disomic strain 5912-SD4 described in Figure 5A using the integration plasmids listed in column 1 and described in WERTMAN *et al.* 1992. The alleles marked in red caused elevated chromosome loss as reflected by the calculated average loss frequencies reported in column four and the standard deviations reported in column five. Note that integration plasmids for *act1-108* and *act1-136* have subsequently been found to each harbor an additional mutation (VIGGIANO *et al.* 2010) and are indicated by an asterisk.
