# Supplementary material for: Actin Dosage Lethality Screening in Yeast Mediated by Selective Ploidy Ablation Reveals Links to Urmylation/Wobble Codon Recognition and Chromosome Stability
Source: G3 (Bethesda). 2013 Mar 1;3(3):553–61. doi: 10.1534/g3.113.005579 (PMC3583461; doi:10.1534/g3.113.005579)
Supplement: Supporting Information [file supp_3.3.553_FigureS1.pdf]

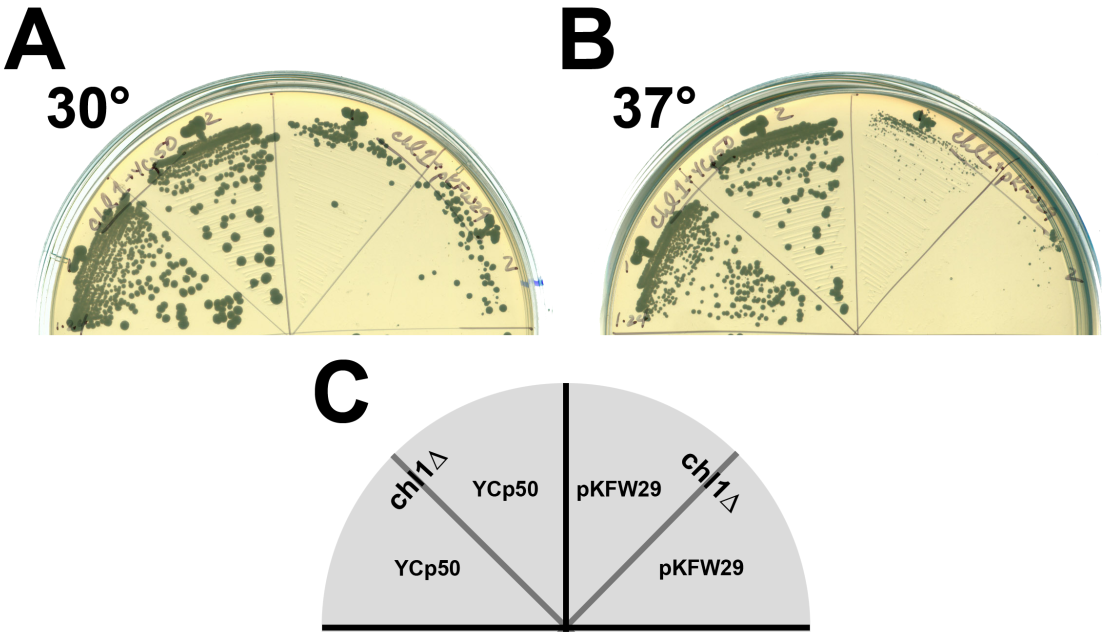

Figure S1 Actin over-expression is toxic to *chl1Δ* cells. A *chl1Δ* strain was transformed with the control plasmid YCp50 or the actin expression plasmid pKFW29, the transformants were streaked on plates according to the key (C) and incubated at 30°C (A) and 37°C (C).
