# Supplementary material for: Actin Dosage Lethality Screening in Yeast Mediated by Selective Ploidy Ablation Reveals Links to Urmylation/Wobble Codon Recognition and Chromosome Stability
Source: G3 (Bethesda). 2013 Mar 1;3(3):553–61. doi: 10.1534/g3.113.005579 (PMC3583461; doi:10.1534/g3.113.005579)
Supplement: Supporting Information [file supp_3.3.553_TableS1.pdf]

**Table S1 Yeast Strains.** All strains used in this study were isogenic with BY4741 except W8164-2B (W303), BBY181, LMMY1 and LMMY3 (S288C) and 5903SD-3 (A364a).

| Name | Source | Genotype |
|---|---|---|
| W8164-2B | R. Rothstein | *MATa CEN1GCS CEN2GCS CEN3GCS CEN4GCS CEN5GCS CEN6GCS CEN7GCS CEN8GCS CEN9GCS CEN10GCS CEN11GCS CEN12GCS CEN13GCS CEN14GCS CEN15GCS CEN16GCS ADE2 can1-100 his3-11,15 leu2-3,112 LYS2 met17 trp1-1 ura3-1 RAD5* |
| LMMY1 | This study | W8164-2B + [pLMM1] |
| LMMY3 | This study | W8164-2B + [pLMM3] |
| BY4741 | SGD | *MAT***a** *his3Δ1 leu2Δ0 ura3Δ0 met15Δ* |
| 5903SD-3 | D. Burke | *MATα ade5/+ lys5-H1/+ cyh2/+ +/aro2-H1 leu1-1/+ trp5-H1/+ +/ade6 ade3/+ ade2-1 his3 Δ1 ura3-52 trp1-289 leu2-3,112 can1* |
| BBY181 | This study | *MAT***a** *ura3-52 his3Δ200 leu2Δ1 trp1Δ63 ssk1Δ::hgh* |
